# Impact of natural salt lick on the home range of *Panthera tigris* at the Royal Belum Rainforest, Malaysia

**DOI:** 10.1038/s41598-021-89980-0

**Published:** 2021-05-19

**Authors:** Bryan Andrew Lazarus, Azlan Che-Amat, Muhammad Muzammil Abdul Halim Shah, Azwan Hamdan, Hasliza Abu Hassim, Farina Mustaffa Kamal, Tengku Rinalfi Putra Tengku Azizan, Mohd Hezmee Mohd Noor, Noordin Mohamed Mustapha, Hafandi Ahmad

**Affiliations:** 1 Department of Veterinary Preclinical Sciences, Faculty of Veterinary Medicine, Universiti Putra Malaysia, 43400 UPM Serdang, Selangor Darul Ehsan Malaysia; 2 Department of Veterinary Clinical Studies, Faculty of Veterinary Medicine, Universiti Putra Malaysia, 43400 UPM Serdang, Selangor Darul Ehsan Malaysia; 3 Department of Veterinary Pathology and Microbiology, Faculty of Veterinary Medicine, Universiti Putra Malaysia, 43400 UPM Serdang, Selangor Darul Ehsan Malaysia; 4 Laboratory of Sustainable Animal Production and Biodiversity, Institute of Tropical Agriculture and Food Security, Universiti Putra Malaysia, 43400 UPM Serdang, Selangor Darul Ehsan Malaysia; 5 University Agriculture Park, Universiti Putra Malaysia, 43400 UPM Serdang, Selangor Darul Ehsan Malaysia

**Keywords:** Systems biology, Zoology, Ecology

## Abstract

Natural salt lick (*sira*) is a strategic localisation for ecological wildlife assemblage to exhibit geophagy which may act as a population dynamic buffer of prey and predators. Undoubtedly, many agree that geophagy at natural licks is linked to nutritional ecology, health and assembly places facilitating social interaction of its users. Overall, natural salt licks not only save energy of obtaining nutrient leading to health maintenance but also forms the basis of population persistence. The Royal Belum Rainforest, Malaysia (Royal Belum) is a typical tropical rainforest in Malaysia rich in wildlife which are mainly concentrated around the natural salt lick. Since this is one of the most stable fauna ecology forest in Malaysia, it is timely to assess its impact on the Malayan tiger (*Panthera tigris*) home range dynamics. The three-potential home ranges of the Malayan tiger in this rainforest were selected based on animal trails or foot prints surrounding the salt lick viz (e.g. *Sira Kuak* and *Sira Batu; Sira Rambai* and *Sira Buluh* and *Sira Papan*) as well as previous sightings of a Malayan tiger in the area, whose movement is dependent on the density and distribution of prey. Camera traps were placed at potential animal trails surrounding the salt lick to capture any encountered wildlife species within the area of the camera placements. Results showed that all home ranges of Malayan tiger were of no significance for large bodied prey availability such as sambar deer (*Rusa unicolor*), and smaller prey such as muntjacs (*Muntiacus muntjac*) and wild boar (*Sus scrofa*). Interestingly, all home range harbour the Malayan tiger as the only sole predator. The non-significance of prey availability at each home range is attributed to the decline of the Malayan tiger in the rainforest since tigers are dependant on the movement of its preferred prey surrounding natural salt licks. Thus, the information from this study offers fundamental knowledge on the importance of prey-predator interaction at salt lick which will help in designing strategy in rewilding or rehabilitation programs of the Malayan tiger at the Royal Belum Rainforest.

## Introduction

Natural salt licks are mineral rich deposits that play a significant role in the ecosystem of the tropical rainforest. Similarly, the importance of salt lick in terms of physiological well-being of animals of plant-based diets is well documented^1–6^ and utilization should not be affected by sex and age categories. In addition, topography has been suggested to affect the accessibility of certain prey to utilize the salt lick due to the changes in mineral content of the salt lick area^2,7,8^.

Salt licks are a determinant of herbivore density in the rainforest, which in turns influence the density and distribution of predators^9–12^. Predators hunt species according to the foraging theory with preference for large bodied prey which posed the least risk and minimal investment for energy^12,13^. Thus, predators play an important role towards balancing the natural ecosystem^14^ and exerting a profound effect on vegetation densities and communities of small vertebrate^7^.

Soil consumption at natural licks is linked to the nutritional ecology and/or health of the users. Other than nutritional benefits, the salt licks also function as animal-assembly venues which facilitates social encounters^15,16^. Therefore, the existence of natural licks in a particular habitat may reduce the costs of obtaining adequate nutrition, and/or maintaining health; and thus, may be fundamental to population persistence.

The Royal Belum Rainforest in Malaysia has been established as one of habitat for Malayan tiger and houses majority of others fauna species including the barking deer or muntjac (*Muntiacus muntjac*), sambar deer (*Rusa unicolor*), Malayan sun bear (*Helarctos malayanus*), Malayan gaur (*Bos gaurus*) and Asian elephant (*Elephas maximus*)^1,2,17,18^. In Malaysia, large bodied prey species such as the sambar deer, and smaller prey like the muntjac and wild pig (*Sus scrofa*) have been described as the key ecological determinant of predators such as the Malayan tiger density and their potential habitat^19,20^. However, prey populations in the tropical rainforests are known to be decreasing due to several factors such as widespread poaching, legal hunting for consumption and deforestation for timber^21,22^. Inevitably, these factors contributed towards the dilution of prey abundance over a large area in the rainforest, affecting the viability of the predators such as the Malayan tiger. Despite advances in the understanding of ecological factors determining prey-predator interactions, no study has investigated how salt lick and prey availability influences the home-range of the Malayan tiger in Royal Belum Rainforest.

Therefore, the aim of this study is to identify the dynamics of prey species at the natural salt lick influencing the home range and possibly the declining numbers of the Malayan tiger at the Royal Belum Rainforest. Data generated from this study will unveil the current distribution of Malayan tigers near the salt licks at Royal Belum Rainforest which will enhance strategic measures in rewilding or rehabilitation of the Malayan tiger.

## Methodology

### Study site

**Figure 1:**
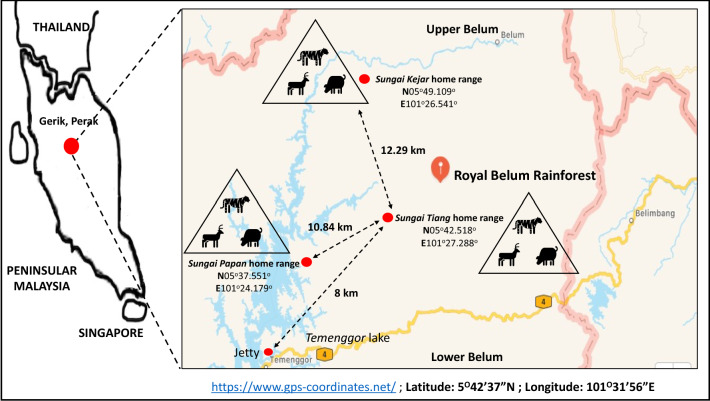
The location of the Royal Belum Rainforest, Peninsular Malaysia and the tracked home range of tigers around natural salt licks. Map details: (1) Peninsular Malaysia: The map was illustrated by the author of this manuscript. (2) Royal Belum Rainforest: Generated from GPS Coordinate (https://www.gps-coordinates.net/); Latitude: 5°42′37″N; Longitude: 101°31′56″E.

The study was conducted from August 2017 to August 2020 at the Royal Belum Rainforest, Gerik Perak, which is located on the central-north of Peninsular Malaysia. This 130-million-year-old tropical rainforest is located at latitude: 5^o^42′37″ N and longitude: 101^o^31′56″ E which is covered by 2990 km^2^ of lowlands and hill dipterocarp virgin forests^23^. This rainforest is rich in flora and fauna which are characteristics of a typical rainforest of Peninsular Malaysia^24^. The forest is divided into two areas, the Upper Belum area, which stretches to the Malaysia-Thailand border and the Lower Belum area covered by the Temenggor Lake (Fig. 1).

Permit of data sampling in the Royal Belum Rainforest was approved by the Perak State Park, Malaysia, and Department of Wildlife National Parks (PERHILITAN) Peninsular Malaysia. Three sampling areas or home ranges were chosen based on the precision of ecological objectives and responses metrics such as wildlife relative abundance, presence of preferred prey (e.g. sambar deer, muntjac and wild pig) utilizing the saltlicks and the sighting of a predatory species (e.g. the Malayan tiger) in the area. The information above was obtained from discussions with the local rangers and local indigenous people who are well versed on the forest ecology. Malayan tigers are generally solitary animals whose movement is dependent on its prey, thus the study assumes that areas containing salt licks might be home ranges of an individual Malayan tiger. The first sampling area at *Sungai Tiang* is located approximately 8 km from the jetty at the Lower Belum, Complex (Fig. 1). Three salt licks namely *Sira Kuak, Sira Batu* and *Sira Tanah* were identified at *Sungai Tiang*. The second, at *Sungai Kejar,* located approximately 12 km from *Sungai Tiang* and is consisted of two salt licks, namely *Sira Rambai* and *Sira Buloh.* The third, at *Sungai Papan*, has only one large salt lick which is *Sira Papan*.

### Camera trap setting

A total of 66 camera traps (LTL Acorn 5210) were placed at strategic locations to identify the frequency of wildlife appearances at all studied salt licks. The number of cameras necessary for monitoring of wildlife at surrounding area of salt licks was in accordance to the size of sample area between 20 and 92 m^2^^2,25^. Each salt lick was tracked by three camera traps, whereas other eight camera traps were placed in areas around the salt lick to enhance the potential of capturing images of the tiger. The placement of the camera was also based on recommendations given by the native’s experts who always encounter tiger footprints around the vicinity of the salt lick.

**Figure 2:**
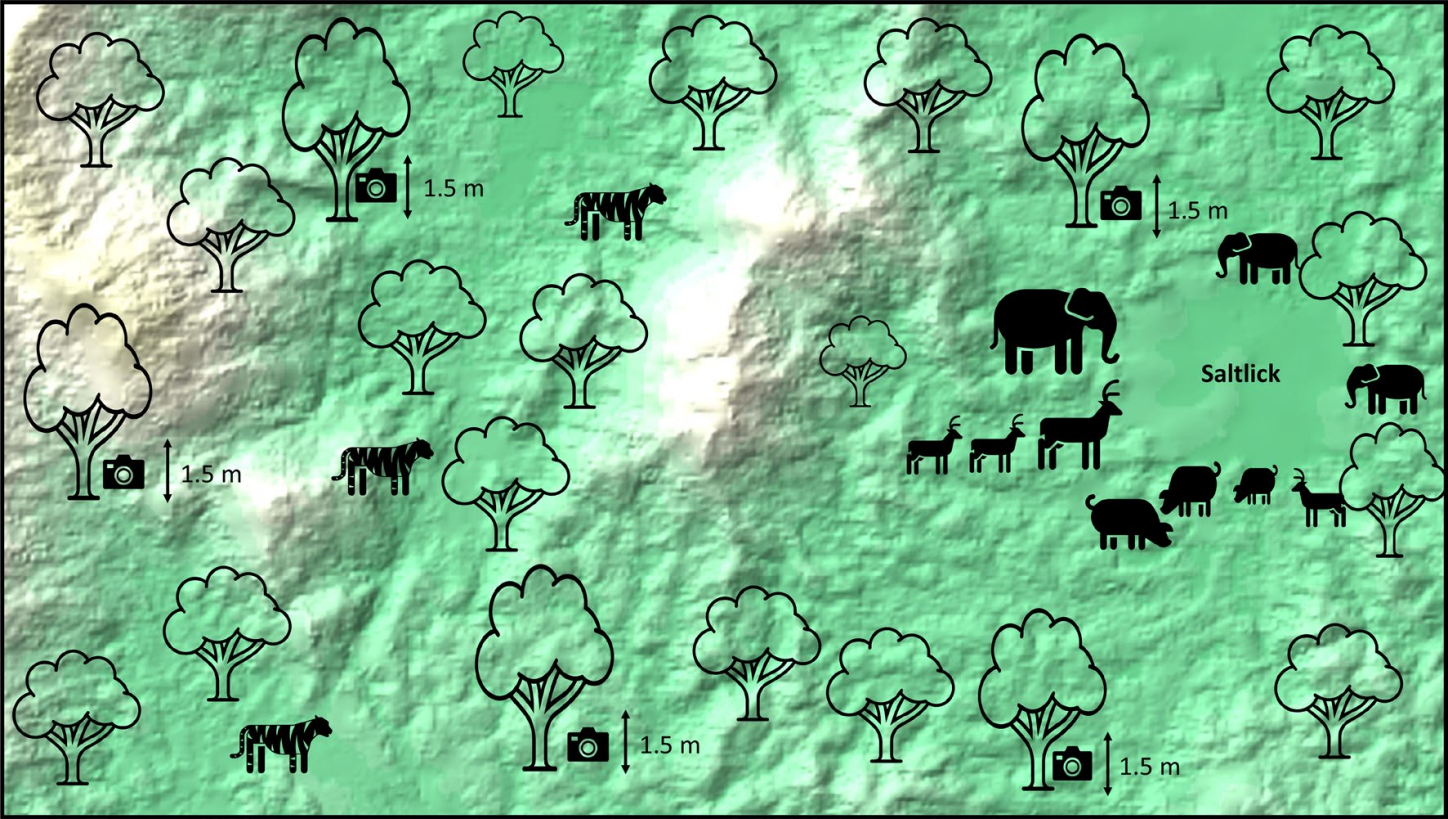
The placement of cameras around a natural salt licks in the Royal Belum Rainforest. Map details: The background featuring a DEM-based image is from the ArcGIS software application (https://www.esri.com/en-us/arcgis/products/arcgis-desktop/overview) with GPS reference coordinate; Latitude: 5°42′37″N; Longitude: 101°31′56″E.

Cameras at saltlicks were placed on various trees around saltlicks directed towards animal paths heading towards the salt licks or drinking sites. The cameras at saltlicks were aimed from a further distance to incorporate a broader angle to obtain a larger view on saltlicks. This was done to obtain a better view for species identification. In regards to the predators, the cameras were placed about 1.5 m off the ground (to commensurate the average height of a tiger) and the cameras were immobilized using bungee cords wrapped around the tree (Fig. 2). Cameras were targeted at wildlife trails 10–50 m outside the saltlicks. Large pathways were incorporated to increase the probability of obtaining an image of a species outside saltlicks.

Each 66-gigabyte memory card (SanDisk, Western Digital) camera is charged with eight AA batteries (Energizer MAX, Energizer Holdings Inc.). The batteries and memory card has a span of approximately two months depending on the frequency of captured images and footages of any moving of objects. The camera is also equipped with infrared sensors that will trigger recording of images and footages upon any movement of animals on a 20 s short video modes. New set of batteries and memory card were replaced 8 times throughout the 36 months duration of the study.

The study was conducted in a gazetted forest, where only those with an authorised permit are allowed entry to minimised theft. However, during the 36 months of sampling, only 1 out of the 6 cameras was stolen and disposed off at *Sira Papan*. The ditched camera devoid of the memory card was found about 20 m from the initial placement. Nevertheless, although about 50% of the installed cameras were either destroyed by elephants or being weathered by humidity, the data from the memory cards were intact.

### Malayan tiger identification

This study attempts to identify the captured images of Malayan tigers. Several established methods have been described for identification of adult tigers in the wild using stripes^26^, pugmarks^27^, DNA^28^, and smell^29^. This study opted for the stripe identification, which is the most common method used for captured image identification of the tigers in the wild^26,30,31^.

### Data recording by the camera trap

All data from the memory cards were played back on Microsoft Windows media player, and the images were tabulated using an ethogram procedure according to the species and number of sightings at each salt lick. Technically, animals of the same species captured within a 30-min period were counted as one individual. Multiple animals of the same species in a single frame were also counted as one individual.

## Statistical analysis

Data obtained from the studies were analysed with MedCalc statistical software version 19.0.4 using one-way analysis of variance (ANOVA) with repeated measures followed by post hoc least significant difference test. All data were expressed as mean ± SD and only *P*-value of less than 0.05 was considered as significant.

## Results

Table 1 shows the number of different species at three different of home range; *Sungai Tiang, Sungai Papan* and *Sungai Kejar* at Royal Belum Rainforest, Gerik Perak. There is no significant different on the prey availability (e.g. muntjac, sambar deer and wild boar) and presence of predators such as the Malayan tiger at all home ranges.

**Table 1:** The number of sightings of different species at the salt licks in the Royal Belum Rainforest (mean ± SD).

Wildlife (species)	Home range/salt lick					
	*Sungai Tiang*			*Sungai Kejar*		*Sungai Papan*
	*Sira Kuak*	*Sira Tanah*	*Sira Batu*	*Sira Bukit*	*Sira Rambai*	*Sira Papan*
Malayan tiger (*Panthera tigris jaksoni*)	0.25 ± 0.25	0.00 ± 0.00	0.00 ± 0.00	0.25 ± 0.25	0.00 ± 0.00	0.75 ± 0.25
Muntjac (*Muntiacus muntjac*)	47.7 ± 40.19	25.0 ± 2.00	21.0 ± 0.82	15.8 ± 2.22	15.0 ± 4.03	23.5 ± 7.31
Sambar (*Rusa unicolor*)	7.8 ± 6.34	21.0 ± 1.63	8.2 ± 1.00	5.8 ± 1.30	7.4 ± 3.93	3.2 ± 1.46
Wild hog/boar (*Sus scrofa*)	6.8 ± 3.42	7.2 ± 2.30	8.2 ± 2.03	25.2 ± 5.64	1633 ± 4.05	23.0 ± 10.29
Malayan gaur (*Bos gaurus hubbacki*)	2.5 ± 1.56	0.0 ± 0.00	0.0 ± 0.00	6.8 ± 2.18	5.8 ± 1.38	0.0 ± 0.00
Asian elephant (*Elephas maximas*)	0.25 ± 0.25	2.0 ± 0.82	16.0 ± 2.10	2.0 ± 0.71	2.8 ± 0.25	0.75 ± 0.48
Malayan tapir (*Tapirus indicus*)	0.25 ± 0.25	2.0 ± 0.82	7.0 ± 1.00	0.0 ± 0.00	0.0 ± 0.00	0.75 ± 0.75
Malayan sun bear (*Helarctos malayanus malayanus*)	0.0 ± 0.00	0.0 ± 0.00	0.0 ± 0.00	0.0 ± 0.00	0.0 ± 0.00	0.25 ± 0.25

Table 2 shows the species, salt licks location and frequency of sighting during the study period. Muntjac and wild boar are two species with higher frequency of sightings at the salt licks.

**Table 2:** The species, salt licks location and frequency of sighting during the study period.

Wildlife (species)	Frequency of sightings based on species at the salt licks			
	*Sungai Tiang*	*Sungai Papan*	*Sungai Kejar*	
Malayan tiger (*Panthera tigris jacksoni*)	1	3	3	0
Muntjac (*Muntiacus muntjac*)	138	82	160	134
Sambar deer (*Rusa unicolor*)	39	16	55	73
Wild pig (*Sus scrofa*)	36	133	204	139

The image of selected wildlife at different home range spotted by the camera traps according to the date and time as stated in the Supplementary Fig. S1–S3. It was revealed that the prey species were either grazing, foraging and exhibiting geophagy at and surrounding natural the salt licks. This includes wild hog either alone or as a passel, muntjac, sambar deer and gaur.

The stripe pattern and close-up image used for identification of a tiger is tabulated in Table 3. Although it was unfortunate that captured view of images is beyond control and could not be standardised, clearly all four Malayan tigers donned different stripe pattern.

**Table 3:**
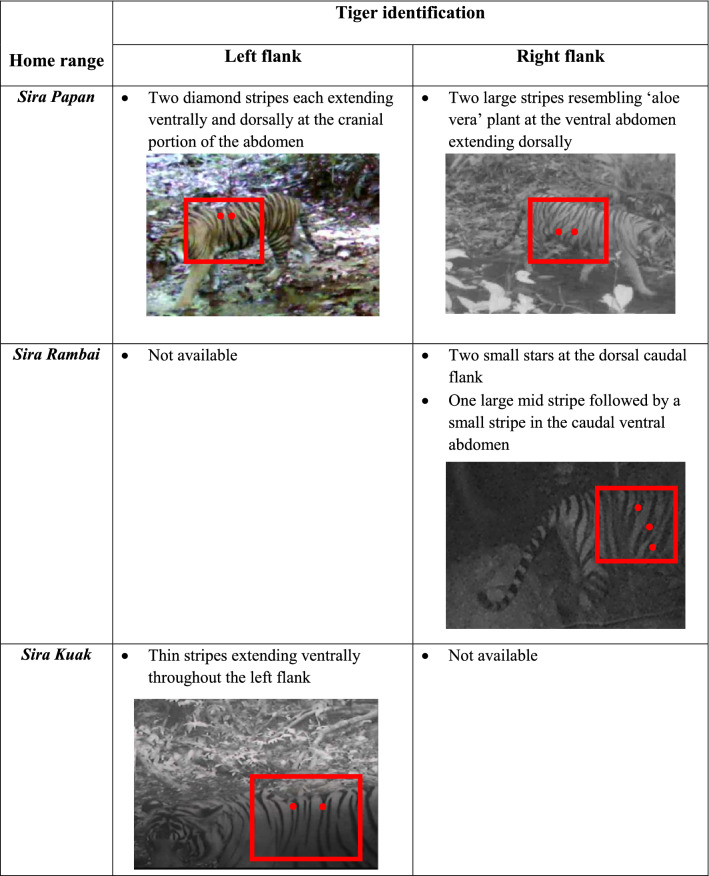
Image identification of Malayan tiger spotted at the Royal Belum Rainforest.

## Discussion

It was documented that the home range for muntjac^32^, sambar deer^33^, wild hog^34^, gaur^35^ and tiger^36^ are 0.6–1.68, 2.4–11.8, 0.6–48.3, 32–169 and 70–294 km^2^, respectively. Based on the stated home range data, the prey species found in the study presented here showed do overlap or provide evidence of their likely encounters with the Malayan tiger. Likewise, it is very likely that these tigers were also “resident” predators in this locality.

In this study, the presence of prey such as muntjac, sambar deer and wild hog are widespread in all home range at the Royal Belum Rainforest. Surprisingly, this contradicts earlier findings denoting their lowest in Malaysia rainforest^37^. It is likely that the latter findings was conducted in a different geographical location (National Park) where activities favouring prey reduction were much more intense^18,21^. However, this finding confirmed the greater availability of prey population in this locality based on salt lick fauna surveillance^2^. Thus, the abundant availability of prey at these salt licks renders a diet selection for the predator which is a vital factor towards equilibrium prey-predator dynamics in the tropical rainforest.

The comparable frequency of prey visiting the natural salt licks between all sites (e.g. *Sungai Tiang*, *Sungai Kejar* and *Sungai Papan*) strengthen the fact that these species are attracted to this site due to the functionality of salt licks. As long as salt licks are functional in providing minerals, differences in herbivore population around natural salt licks is unexpected unless it is influenced by external factors such as an increase in number of predators. The inference made from this study that only one carnivore was documented around each salt lick explains the absence of a top-down regulator affecting herbivore occurrence in that area.

The spotting of only one adult Malayan tiger at each home range further support tigers to be solitary and territorial, and conformed to their documented home range (7–294 km^2^). Likewise, this finding is also denotes that despite the failure in getting a full stripe profile of each spotted tiger, they were actually a different individual.

The preferred prey of the Malayan tiger is namely the sambar deer, wild hog and muntjac^37–40^ that were frequently noted around the salt licks indicated the impact of salt licks as an invaluable hunting grounds for this species. This could indicate that the Malayan tiger localises its home range, and roams around natural salt licks with confidence of capturing prey of its choice^8,41^.

The findings from the study presented here cast the pivotal role of predators stabilising the fauna ecosystem by influencing the population of prey communities in the salt lick area^4,42^. Thus, the conservation of natural salt lick is not only important for prey, but also has direct bearing to the longevity and survival of the Malayan tigers.

Undoubtedly, this study showed the dependence of the Malayan tigers in the Royal Belum Rainforest on the density and abundance of prey species. Similarly, the due to their dependence on natural salt licks, prey species tend to habituate at the natural salt licks which directly invoke the presence of tiger to this area. We believe that the ‘home-range’ of the territorial carnivores are based around clusters of natural salt licks located in the tropical rainforest. In fact, the long distance between different home range (as shown in Fig. 1) combined with a low prey abundance of tropical rainforests, will indirectly lead to the dispersal and isolation of our already severely debilitated number of adult tigers^42^. In addition, large home ranges, wide dietary breadth and dense forested habitats have precluded precise quantification of diet constituents and accurate sampling techniques for determining available prey base in most of the tiger’s geographical range^39,43^.

**Figure 3:**
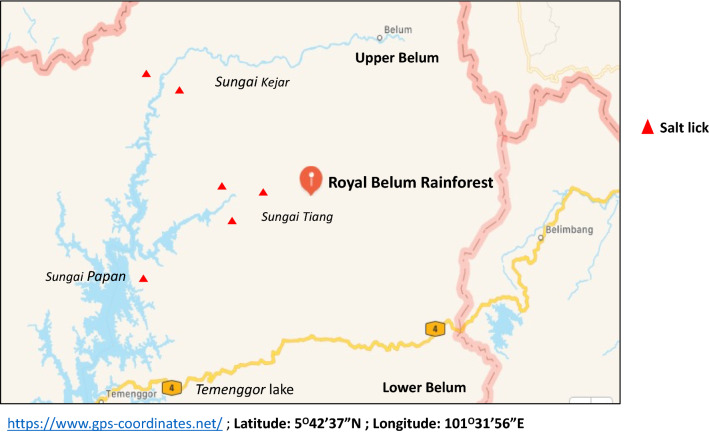
The distribution of salt licks at the Royal Belum Rainforest. Map details: Royal Belum Rainforest: Generated from GPS Coordinate (https://www.gps-coordinates.net/); Latitude: 5°42′37″N; Longitude: 101°31′56″E.

Hence, this study puts a postulate that tigers in the Royal Belum Rainforest are too dispersed and isolated from one another to maintain a healthy, breeding, and genetically diverse population. Tigers are localising around areas of prey concentration at natural salt licks; thus, they do not need to roam large areas of tropical rainforests in search of prey. This puts them in dispersed areas, as salt licks are located in randomly distributed locations throughout the tropical rainforests (Fig. 3). Besides low prey populations, forest fragmentation and continued poaching are leading towards low occurrence of tigers in the same areas, and isolation of tigers in fragmented forests with no way to maintain the population^37,40^. It appears that poaching activity exist out within the Royal Belum Rainforest. In our study, we encountered evidence of poaching in the form of a Malayan gaur which was missing a hoof in the hind right limb (Supplementary Fig. S4). This injury could have been acquired from a snare meant for animals like the Malayan tiger or sun bears which are sought after for traditional medicine^21^.

## Supplementary Information

Supplementary Information.

## Data Availability

The location and statistical data used in this study are available included within this article. Some of confidential data are restricted by the Royal Belum State Park in order to protect the endangered species located within the area of study. Data are available from the corresponding author, Associate Professor Dr. Hafandi Ahmad (hafandi@upm.edu.my) for researchers who meet the criteria for access to confidential data.
