# Supplementary material for: Impact of natural salt lick on the home range of Panthera tigris at the Royal Belum Rainforest, Malaysia
Source: Sci Rep. 2021 May 19;11:10596. doi: 10.1038/s41598-021-89980-0 (PMC8134436; doi:10.1038/s41598-021-89980-0)
Supplement: Supplementary file 1 — Supplementary Information. [file 41598_2021_89980_MOESM1_ESM.docx]

**Supplementary Information**

**Impact of natural salt lick on the home range of *Panthera tigris* at the Royal Belum Rainforest, Malaysia**

**Table of contents**

**I. Supplementary figures**

1. Supplementary Figure 1

2. Supplementary Figure 2

3. Supplementary Figure 3

4. Supplementary Figure 4

** Corresponding author:*

Assoc Prof Dr Hafandi Ahmad

Department of Veterinary Preclinical Sciences

Faculty of Veterinary Medicine

Universiti Putra Malaysia

43400 UPM Serdang, Selangor Darul Ehsan, MALAYSIA

Email : [hafandi@upm.edu.my](mailto:hafandi@upm.edu.my)

Tel : +603-8609- 3416 ; Fax : +603- 9769-1971


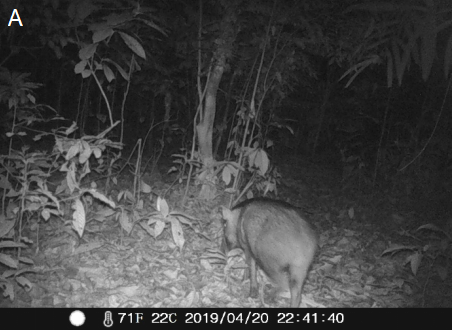

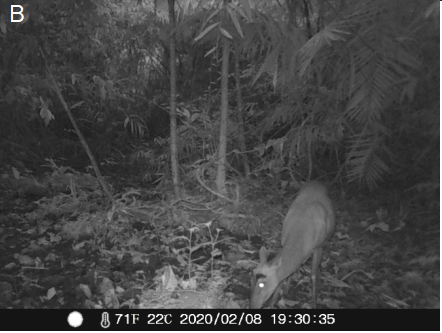

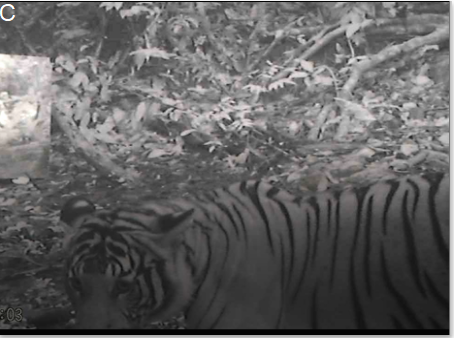

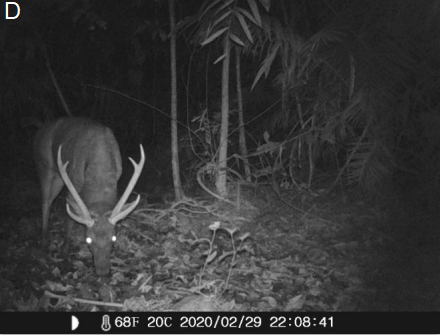


**Supplementary Figure 1 |** Photograph of a (A) wild boar and (B) Muntjac captured on 20^th^ March 2020 (10.41pm) and 8th^th^ of February 2020 (7.30pm), respectively at *Sungai Tiang* home range. The image of a (C) Malayan tiger in a clearing 20m from the saltlick on the 29^th^ May 2017 (11.13am) and (D) Sambar deer was captured on the 29^th^ of February 2020 (10.08pm) at *Sungai Tiang* home range. Photographs were taken by Hafandi Ahmad ([hafandi@upm.edu.my](mailto:hafandi@upm.edu.my)).


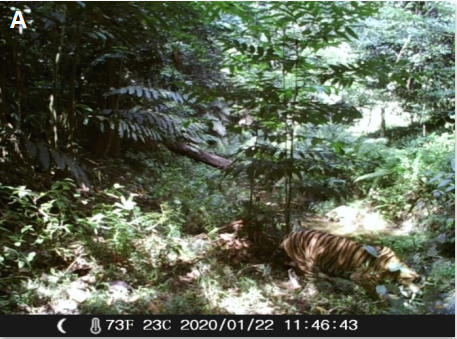

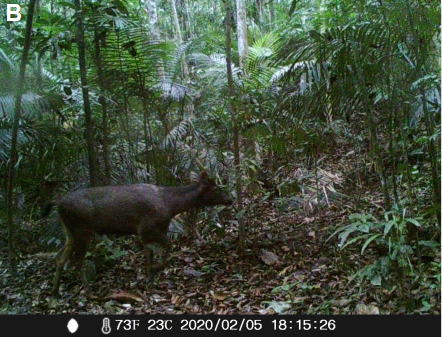

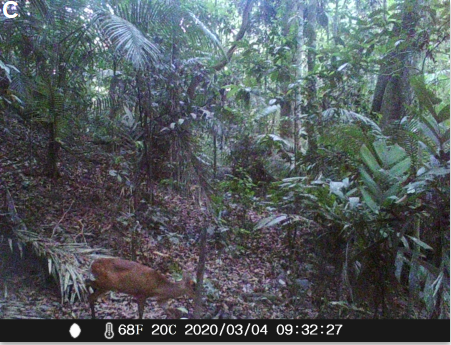

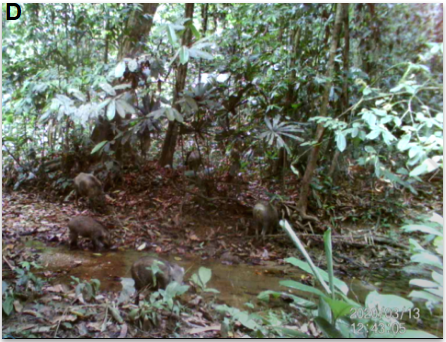


**Supplementary Figure 2 |** Photograph of an almost full portrait of a (A) Malayan tiger and (B) Sambar deer seen on 22^nd^ January 2020 (11.46am) and 5^th^ May 2020 (18.15pm), respectively at *Sungai Papan* home range. The image of a (C) Muntjac and (D) passel spotted on the 4^th^ of April 2020 (9.32 am) and 22^nd^ January 2020 (9.19am), respectively at *Sungai Papan* home range. Photographs were taken by Hafandi Ahmad ([hafandi@upm.edu.my](mailto:hafandi@upm.edu.my)).


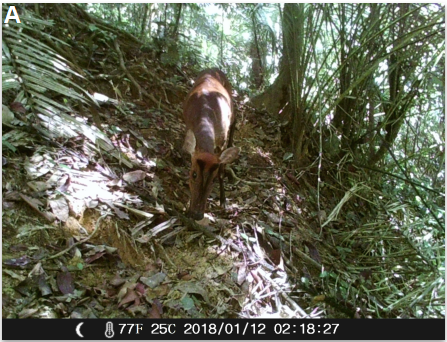

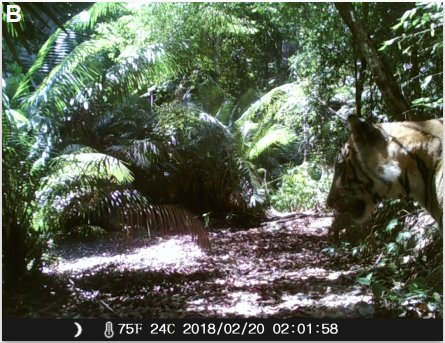

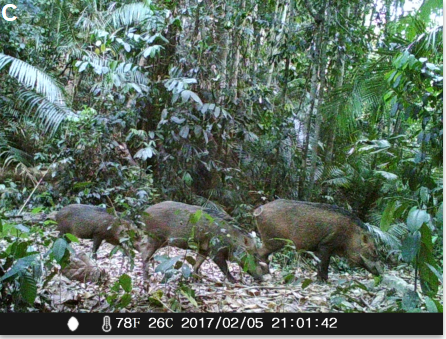

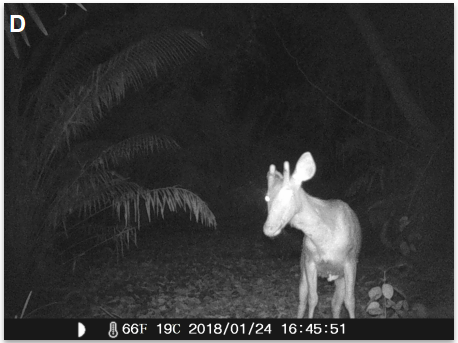


**Supplementary Figure 3 |** Photograph a (A) Muntjac and (B) Malayan tiger captured on the 12^th^ of January 2018 (9.01am) and 20^th^ of February 2019 (14.01pm), respectively at *Sungai Kejar* home range. The image of a (C) passel and (D) Samba captured on 5^th^ of February 2017 (9.01am) and 24^th^ of January 2018 (22.08pm), respectively at *Sungai Kejar* home range. Photographs were taken by Hafandi Ahmad ([hafandi@upm.edu.my](mailto:hafandi@upm.edu.my)).


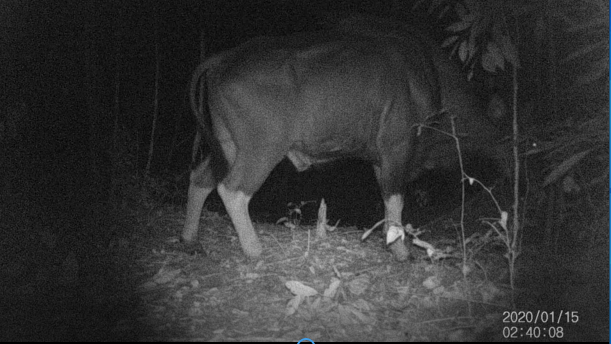


**Supplementary Figure 4 |** A wild gaur documented on our cameras missing its hind right hoof. Photographs were taken by Hafandi Ahmad ([hafandi@upm.edu.my](mailto:hafandi@upm.edu.my)).
